# Supplementary material for: Systematic evaluation of medication adherence determinants across 137 active substances on population-level real-world health data
Source: Commun Med (Lond). 2026 Mar 9;6:237. doi: 10.1038/s43856-026-01515-8 (PMC13103320; doi:10.1038/s43856-026-01515-8)
Supplement: Supplementary file 2 — Supplementary material [file 43856_2026_1515_MOESM2_ESM.pdf]

Systematic evaluation of medication adherence determinants across 137 ingredients on population-level real-world health data

Kerli Mooses[a], Marek Oja[a], Maria Malk[a], Helene Loorents[a], Maarja Pajusalu[a], Nikita Umov[b], Sirli Tamm[a], Johannes Holm[a], Hanna Keidong[a], Taavi Tillmann[b], Sulev Reisberg[a], Jaak Vilo[a], Raivo Kolde[a]

[a] Institute of Computer Science, University of Tartu, Narva mnt 18, Tartu, Estonia

[b] Institute of Family Medicine and Public Health, University of Tartu, Ravila 19, 50411 Tartu, Estonia

Supplementary Table 1. Study design, patient inclusion and exclusion criteria, time periods and covariates in different models performed in the study.

Covariates of interest		Factors influencing medication adherence / Calculation of IMAS (Fig. 1 step 2)	IMAS across drug classes (Fig. 1 step 3.1.)			IMAS temporal stability (Fig. 1, step 3.2.)	IMAS impact on health outcomes (Fig. 1, step 3.3.)	
			CVD* model	Other active substances without IMAS	Other active substances with IMAS		LMM to calculate IMAS	Survival analysis on hospitalisation and incidence
Demographical (fixed-effect)	Gender	X	X	X	X	X	X	X
	Age	X	X	X	X	X	X	
	Year of birth							X
Health-related (fixed-effect)	Body mass index	X	X	X	X	X	X	
	Hospitalization	X	X	X	X	X	X	
	Depression	X	X	X	X	X	X	
	Dementia or mental retardation	X	X	X	X	X	X	
	N of diagnoses per year	X	X	X	X	X	X	
	N of active substances administered per year	X	X	X	X	X	X	
	Comorbidity	X	X	X	X	X	X	
Medication-related (fixed-effect)	Active substance	X	X	X	X	X	X	
	Administration route	X	X	X	X	X	X	
	Consecutive year of administration	X	X	X	X	X	X	
	Co-occurrence of active substance and related diagnosis	X	X	X	X	X	X	
Person (random-effect)		X	X	X	X	X	X	
IMAS extracted from LMM					X (from CVD model)			X
Active substances included		137	Associated with CVD	Non-CVD	Non-CVD	137	137	
Included subjects		Subjects who have CMA calculated for at least for one active substance under observation	Subjects who have CMA calculated for active substance associated with CVD			Subjects who have CMA calculated for at least for one active substance under observation	Subjects who have CMA calculated for at least for one active substance under observation	
Time period		Across 2012-2019	Across 2012-2019			On yearly basis 2012-2019	Across 2012-2016	
Study design		Cross-sectional study	Cross-sectional study			Staggered cohort study	Cohort study	

*CVD – cardiovascular diseases (hypertension (I10-I15), arrhythmias (I46-I49), heart failure (I50), ischemic heart disease (I20-I25), other diseases of the circulatory system (I67-I70))

Supplementary Table 2. The number of cases included into the Cox proportional hazards model to estimate incidence and hospitalisation for selected diseases.

Cause	ICD-10 codes	Incidence		Hospitalisation	
		Cases	Risk population	Cases	Risk population
Disorders of thyroid gland	E00-E07	1442	39772		
Diabetes mellitus	E10-E14	1191	39781		
Disorders of lipoprotein metabolism and other lipidaemias	E78	2929	31728		
Depression	F32, F33	1543	38595		
Neurotic disorders	F40-F48	2245	36492		
Anemia	D50-D64	2728	42485		
Ischemic heart disease	I20-I25	2252	37801		
Myocardial infraction	I21-I23			508	47064
Cardiac valve disorders	I34-I37	543	46561		
Cardic arrhythmias	I46-I49	3115	37304	661	46671
Heart failure	I50	2603	38783	320	47596
Cerebral ischemia/stroke	I60-I64, I69, G45	1607	43828	882	46536
Atherosclerosis/peripheral arterial occlusive disease	I65-I67, I70, I73	1898	41970		
Asthma and chronic obstructive bronchitis	J43-J47	1677	40123		
Chronic cholecystitis/ gallstones	K80-K81	1593	44003		
Osteoporosis	M80-M82	985	45580		
Renal failure	N17-N19	1671	46059		
Gout	M10	1608	44137		
Diseases of liver	K70-K77	1094	45678		
Parkinson's disease	G20-G22	251	47271		
Diseases of stomach	K20-K31	4214	28702		
Insomnia	G47, F51	3087	35667		
Dementia	F00-F03, G30, G31	988	46314		
Varicose veins of lower extremities	I83, I87	1496	43891		
Migrane/chornic headache	G43-G44	1137	44205		
Dizziness	H81-H82, R42	2432	41620		
Severe hearing loss	H90-H91	2202	43073		
Aneurysm, thrombosis, embolism	I71, I72, I74, I80, I81, I82, I26	1520	44885		
Injuries	S00-S99	5634	30497	1122	46163
Hip fracture	S72	439	47434	367	47535
Pneumonia	J12-J18	2875	43342	576	47106
Urinary tract infection	N10, N12, N15, N30	2649	39121	114	47759
Gastrointestinal bleeding	K25-K28	1060	44147	166	47676

Supplementary Table 3. The effect of person-specific medication adherence on cause-specific hospitalisation in Cox proportional hazards model.

Disease (ICD-10 code)	N of events	Risk population	Hazard ratio	95% CI	p-value	Sig[1]
Myocardial infraction (I21-I23)	508	47 064	0.43	0.19…0.99	0.046	
Cardiac arrhythmias (I46-I49)	661	46 671	0.76	0.37…1.59	0.470	
Heart failure (I50)	320	47 596	0.63	0.22…1.83	0.398	
Cerebral ischemia/stroke (I60-I64, I69, G45)	882	46 536	0.44	0.23…0.82	0.009	
Injuries (S00-S99)	1122	46 163	0.59	0.34…1.03	0.061	
Hip fracture (S72)	367	47 535	1.03	0.37…2.81	0.961	
Pneumonia (J12-J18)	576	41 106	1.54	0.69…3.46	0.295	
Urinary tract infection (N10, N12, N15, N30)	114	47 759	0.40	0.07…2.24	0.294	
Gastrointestinal bleeding (K25-K28)	166	47 676	1.65	0.36…7.45	0.517	

[1] Bonferroni adjusted p-value: 0.006

Supplementary Table 4. The effect of person-specific medication adherence on cause-specific incidence in Cox proportional hazards model.

Cause	Cases	Risk population	Hazard ratio	95%CI	p-valueSig[1]
Disorders of thyroid gland (E00-E07)	1 442	39 772	0.59	0.36...0.95	0.031
Diabetes mellitus (E10-E14)	1 191	39 781	1.44	0.83...2.51	0.193
Disorders of lipoprotein metabolism and other lipidaemias (E78)	2 929	31 728	0.48	0.34...0.68	< 0.001*
Depression (F32, F33)	1 543	38 595	0.76	0.47...1.22	0.255
Neurotic disorders (F40-F48)	2 245	36 492	0.71	0.48...1.06	0.093
Anemia (D50-D64)	2 728	42 485	0.96	0.67...1.38	0.821
Ischemic heart disease (I20-I25)	2 252	37 801	0.40	0.27...0.59	< 0.001*
Cardiac valve disorders (I34-I37)	543	46 561	0.82	0.36...1.85	0.630
Cardic arrhythmias (I46-I49)	3 115	37 304	0.66	0.47...0.92	0.014
Heart failure (I50)	2 603	38 783	0.85	0.59...1.23	0.395
Cerebral ischemia/stroke (I60-I64, I69, G45)	1 607	43 828	0.45	0.29...0.72	< 0.001*
Atherosclerosis/peripheral arterial occlusive disease (I65-I67, I70, I73)	1 898	41 970	0.27	0.18....0.41	< 0.001*
Asthma and chronic obstructive bronchitis (J43-J47)	1 677	40 123	0.32	0.20...0.50	< 0.001*
Chronic cholecystitis/ gallstones (K80-K81)	1 593	44 003	0.42	0.26...0.66	< 0.001*
Osteoporosis (M80-M82)	985	45 580	0.30	0.17...0.54	< 0.001*
Renal failure (N17-N19)	1 671	46 059	0.82	0.51...1.30	0.394
Gout (M10)	1 608	44 137	1.18	0.74...1.90	0.488
Diseases of liver (K70-K77)	1 094	45 678	0.27	0.15...0.47	< 0.001*
Parkinson's disease (G20-G22)	251	47 271	2.05	0.59...7.08	0.257
Diseases of stomach (K20-K31)	4 214	28 702	0.58	0.44...0.78	< 0.001*
Insomnia (G47, F51)	3 087	35 667	0.45	0.32...0.63	< 0.001*
Dementia (F00-F03, G30, G31)	988	46 314	0.46	0.25...0.84	0.011
Varicose veins of lower extremities (I83, I87)	1 496	43 891	0.30	0.19...0.49	< 0.001*
Migraine/chronic headache (G43-G44)	1 137	44 205	0.36	0.21...0.64	< 0.001*
Dizziness (H81-H82, R42)	2 432	41 620	0.37	0.25...0.54	< 0.001*
Severe hearing loss (H90-H91)	2 202	43 073	0.46	0.31...0.68	< 0.001*
Aneurysm, thrombosis, embolism (I71, I72, I74, I80, I81, I82, I26)	1 520	44 885	0.32	0.20...0.51	< 0.001*
Injuries (S00-S99)	5 634	30 497	0.49	0.38...0.62	< 0.001*
Hip fracture (S72)	439	47 434	0.78	0.31...1.93	0.587
Pneumonia (J12-J18)	2 875	43 342	0.77	0.54...1.09	0.134
Urinary tract infection (N10, N12, N15, N30)	2 649	39 121	0.67	0.47...0.97	0.034
Gastrointestinal bleeding (K25-K28)	1 060	44 147	0.34	0.19...0.60	< 0.001*

[1] Bonferroni adjusted p-value: 0.002
