# Supplementary material for: Systematic evaluation of medication adherence determinants across 137 active substances on population-level real-world health data
Source: Commun Med (Lond). 2026 Mar 9;6:237. doi: 10.1038/s43856-026-01515-8 (PMC13103320; doi:10.1038/s43856-026-01515-8)
Supplement: Supplementary file 3 — Description for Supplementary Data Files [file 43856_2026_1515_MOESM3_ESM.docx]

**Description for Supplementary Data Files**

**Supplementary Data 1. Ingredients included in the analysis**

This file provides the list of active substances included in the study (n = 137), together with the number of calculated continuous multiple interval measures of medication availability (CMA), the number of subjects per ingredient, the most frequent diagnosis recorded on the prescription, the assigned disease group, and the corresponding ICD-10 chapter

**Supplementary Data 2. Average CMA by ingredient**

This file presents the average continuous multiple interval measure of medication availability (CMA) for each active substance, along with corresponding 95% confidence intervals. These values summarise medication adherence at the ingredient level and support between-substance comparisons reported in the main text.

**Supplementary Data 3. Average age by ingredient**

This file reports the mean age and 95% confidence intervals of subjects receiving each active substance. These data describe the age distribution of medication users and provide additional context for interpreting adherence patterns across therapeutic groups.

**Supplementary Data 4. Linear mixed model estimates**

This file contains the full results of the linear mixed model assessing associations between demographic, health-, medication-, and diagnosis-related variables and medication adherence (CMA). Reported parameters include regression estimates, standard errors, 95% confidence intervals, p-values, and indicators of statistical significance after Bonferroni correction. The file also includes ingredient-level effects and ingredient-by-diagnosis interaction terms.
