# Supplementary material for: Systematic evaluation of medication adherence determinants across 137 active substances on population-level real-world health data
Source: Commun Med (Lond). 2026 Mar 9;6:237. doi: 10.1038/s43856-026-01515-8 (PMC13103320; doi:10.1038/s43856-026-01515-8)
Supplement: Supplementary file 4 — Supplementary Data 1. Ingredients included in the analysis [file 43856_2026_1515_MOESM4_ESM.docx]

# **Supplementary data 1.** Ingredients included into the analysis, number of calculated continuous multiple interval measures of medication availability (CMA) measures, number of subjects, and most frequent diagnoses on the prescription

| Ingredient | N of CMAs | N subjects | Disease group | ICD-10 Chapter |
| --- | --- | --- | --- | --- |
| anastrozole | 529 | 205 | Malignant neoplasm of breast (C50) | Neoplasms (C00-D48) |
| letrozole | 877 | 368 |  |  |
| tamoxifen | 747 | 324 |  |  |
| bicalutamide | 971 | 479 | Malignant neoplasm of prostate (C61) |  |
| levothyroxine | 24153 | 5443 | Disorders of thyroid gland (E00-E07) | Endocrine, nutritional and metabolic diseases (E00-E90) |
| methimazole | 1161 | 688 |  |  |
| empagliflozin | 1342 | 672 | Diabetes (E10-E14) |  |
| gliclazide | 8225 | 2150 |  |  |
| glimepiride | 5625 | 1459 |  |  |
| insulin aspart | 4764 | 1167 |  |  |
| insulin detemir | 3386 | 884 |  |  |
| insulin glargine | 4666 | 1195 |  |  |
| insulin glulisine, human | 756 | 179 |  |  |
| insulin lispro | 1621 | 374 |  |  |
| linagliptin | 2328 | 927 |  |  |
| liraglutide | 977 | 335 |  |  |
| metformin | 32311 | 7659 |  |  |
| metoprolol | 69748 | 18179 |  |  |
| saxagliptin | 445 | 170 |  |  |
| sitagliptin | 4208 | 1127 |  |  |
| vildagliptin | 893 | 349 |  |  |
| atorvastatin | 22550 | 7074 | Disorders of lipoprotein metabolism and other lipidaemias (E78) |  |
| fluvastatin | 713 | 218 |  |  |
| pravastatin | 657 | 173 |  |  |
| rosuvastatin | 25418 | 7196 |  |  |
| simvastatin | 11751 | 3035 |  |  |
| donepezil | 759 | 335 | Dementia (F00-F03, G30, G31) | Mental and behavioural disorders (F00-F99) |
| melperone hydrochloride | 1764 | 835 |  |  |
| agomelatine | 1207 | 923 | Psychotic, mood and neurotic disorders (F20-F48, F90) |  |
| aripiprazole | 640 | 235 |  |  |
| bupropion | 1268 | 745 |  |  |
| chlorprothixene | 943 | 307 |  |  |
| citalopram | 2204 | 926 |  |  |
| clozapine | 1042 | 269 |  |  |
| duloxetine | 2842 | 1368 |  |  |
| escitalopram | 8264 | 4176 |  |  |
| fluoxetine | 3496 | 1745 |  |  |
| flupenthixol | 864 | 418 |  |  |
| haloperidol | 2153 | 1003 |  |  |
| methylphenidate | 939 | 420 |  |  |
| mirtazapine | 3834 | 1900 |  |  |
| nortriptyline | 810 | 336 |  |  |
| olanzapine | 1729 | 634 |  |  |
| paroxetine | 2774 | 1140 |  |  |
| promazine | 847 | 327 |  |  |
| risperidone | 1740 | 727 |  |  |
| sertraline | 4203 | 2238 |  |  |
| tianeptine | 3025 | 1861 |  |  |
| trihexyphenidyl | 1435 | 455 |  |  |
| venlafaxine | 2923 | 1326 |  |  |
| vortioxetine | 548 | 414 |  |  |
| benserazide | 1635 | 503 | Parkinson disease (G20) | Diseases of the nervous system (G00-G99) |
| levodopa | 2055 | 579 |  |  |
| pramipexole | 769 | 276 |  |  |
| rasagiline | 804 | 239 |  |  |
| carbamazepine | 3925 | 1247 | Epilepsy (G40-G42) |  |
| lamotrigine | 1047 | 350 |  |  |
| levetiracetam | 454 | 145 |  |  |
| oxcarbazepine | 961 | 279 |  |  |
| valproate | 2526 | 721 |  |  |
| betaxolol | 1270 | 441 | Glaucoma (H40-H42) | Diseases of the eye and adnexa (H00-H59) |
| bimatoprost | 2120 | 733 |  |  |
| brinzolamide | 4839 | 1524 |  |  |
| dorzolamide | 5701 | 1788 |  |  |
| latanoprost | 8627 | 2614 |  |  |
| tafluprost | 1705 | 666 |  |  |
| timolol | 15153 | 4024 |  |  |
| travoprost | 3966 | 1170 |  |  |
| amlodipine | 53205 | 14911 | Hypertension (I10-I15) | Diseases of the circulatory system (I00-I99) |
| atenolol | 1500 | 388 |  |  |
| bisoprolol | 3370 | 1341 |  |  |
| candesartan | 6500 | 1946 |  |  |
| enalapril | 20166 | 5430 |  |  |
| felodipine | 3446 | 920 |  |  |
| fosinopril | 9737 | 2553 |  |  |
| hydrochlorothiazide | 41332 | 10915 |  |  |
| indapamide | 24722 | 8396 |  |  |
| lacidipine | 4852 | 1367 |  |  |
| lercanidipine | 5478 | 1812 |  |  |
| lisinopril | 2326 | 721 |  |  |
| losartan | 7254 | 1911 |  |  |
| moxonidine | 2564 | 1113 |  |  |
| nebivolol | 21924 | 6831 |  |  |
| nifedipine | 1912 | 822 |  |  |
| nitrendipine | 6121 | 2074 |  |  |
| olmesartan | 9696 | 3305 |  |  |
| perindopril | 29351 | 10107 |  |  |
| propranolol | 1705 | 798 |  |  |
| ramipril | 32550 | 10038 |  |  |
| telmisartan | 34302 | 9070 |  |  |
| trandolapril | 1172 | 387 |  |  |
| valsartan | 5482 | 1586 |  |  |
| verapamil | 4085 | 1316 |  |  |
| isosorbide | 6460 | 2078 | Ischemic heart disease (I20-I25) |  |
| trimetazidine dihydrochloride | 9032 | 3522 |  |  |
| amiodarone | 3275 | 1346 | Arrhythmias (I46-I49) |  |
| apixaban | 3471 | 1929 |  |  |
| dabigatran | 2026 | 956 |  |  |
| propafenone | 4708 | 1599 |  |  |
| rivaroxaban | 6706 | 2977 |  |  |
| sotalol | 2186 | 660 |  |  |
| warfarin | 10576 | 2976 |  |  |
| carvedilol | 1548 | 500 | Heart failure (I50) |  |
| digoxin | 7334 | 2453 |  |  |
| furosemide | 4623 | 2321 |  |  |
| spironolactone | 7352 | 3241 |  |  |
| torsemide | 11610 | 5492 |  |  |
| nafronyl | 2245 | 1010 | Other diseases of the circulatory system (I67-I70) |  |
| pentoxifylline | 3834 | 1612 |  |  |
| piracetam | 965 | 591 |  |  |
| albuterol | 9250 | 5330 | Chronic diseases of respiratory system (J43-J47) | Diseases of the respiratory system (J00-J99) |
| formoterol | 8446 | 2989 |  |  |
| glycopyrronium | 1038 | 431 |  |  |
| indacaterol | 911 | 389 |  |  |
| ipratropium | 1766 | 776 |  |  |
| montelukast | 3232 | 1705 |  |  |
| theophylline | 1810 | 752 |  |  |
| tiotropium | 2012 | 812 |  |  |
| amylase | 2384 | 1207 | Diseases of the digestive system (K00-K93) | Diseases of the digestive system (K00-K93) |
| lipase | 2384 | 1207 |  |  |
| mesalamine | 944 | 306 |  |  |
| protease | 2384 | 1207 |  |  |
| ursodeoxycholate | 667 | 343 |  |  |
| hydroxychloroquine | 1869 | 704 | Rheumatoid arthritis and related disorders (M05, M06, M08, M13, M30-35, M45) | Diseases of the musculoskeletal system and connective tissue (M00-M99) |
| leflunomide | 731 | 239 |  |  |
| methotrexate | 2803 | 1081 |  |  |
| sulfasalazine | 1536 | 552 |  |  |
| allopurinol | 11183 | 4164 | Gout (M10) |  |
| febuxostat | 1277 | 745 |  |  |
| alendronate | 3890 | 1366 | Osteoporosis (M80, M81) |  |
| alfuzosin | 2180 | 1214 | Diseases of male genital organs (N40-N51) | Diseases of the genitourinary system (N00-N99) |
| doxazosin | 2220 | 781 |  |  |
| dutasteride | 3836 | 1337 |  |  |
| tamsulosin | 11020 | 3920 |  |  |
| clopidogrel | 4927 | 2428 | Presence of cardiac and vascular implants and grafts (Z95) | Factors influencing health status and contact with health services (Z00-Z99) |
| cyclosporine | 444 | 153 |  |  |
| ticagrelor | 1038 | 796 |  |  |
