# Supplementary material for: Systematic evaluation of medication adherence determinants across 137 active substances on population-level real-world health data
Source: Commun Med (Lond). 2026 Mar 9;6:237. doi: 10.1038/s43856-026-01515-8 (PMC13103320; doi:10.1038/s43856-026-01515-8)
Supplement: Supplementary file 5 — Supplementary Data 2. Average CMA by ingredient [file 43856_2026_1515_MOESM5_ESM.docx]

**Supplementary Data 2.** Average CMA with 95% confidence intervals by ingredients

| Ingredient | Average CMA | 95% CI |
| --- | --- | --- |
| agomelatine | 0.766 | 0.750…0.783 |
| albuterol | 0.423 | 0.414…0.432 |
| alendronate | 0.812 | 0.801…0.823 |
| alfuzosin | 0.733 | 0.720…0.747 |
| allopurinol | 0.727 | 0.720…0.734 |
| amiodarone | 0.781 | 0.770…0.793 |
| amlodipine | 0.796 | 0.793…0.799 |
| amylase | 0.453 | 0.436…0.469 |
| anastrozole | 0.901 | 0.883…0.918 |
| apixaban | 0.834 | 0.825…0.842 |
| aripiprazole | 0.794 | 0.766…0.822 |
| atenolol | 0.790 | 0.768…0.812 |
| atorvastatin | 0.787 | 0.782…0.792 |
| benserazide | 0.825 | 0.807…0.843 |
| betaxolol | 0.786 | 0.765…0.807 |
| bicalutamide | 0.820 | 0.803…0.838 |
| bimatoprost | 0.784 | 0.769…0.798 |
| bisoprolol | 0.768 | 0.756…0.780 |
| brinzolamide | 0.822 | 0.812…0.831 |
| bupropion | 0.797 | 0.781…0.813 |
| candesartan | 0.730 | 0.720…0.740 |
| carbamazepine | 0.719 | 0.704…0.733 |
| carvedilol | 0.776 | 0.757…0.795 |
| chlorprothixene | 0.829 | 0.804…0.853 |
| citalopram | 0.767 | 0.752…0.781 |
| clopidogrel | 0.865 | 0.858…0.872 |
| clozapine | 0.829 | 0.807…0.852 |
| cyclosporine | 0.866 | 0.836…0.897 |
| dabigatran | 0.763 | 0.746…0.780 |
| digoxin | 0.813 | 0.806…0.821 |
| donepezil | 0.805 | 0.781…0.828 |
| dorzolamide | 0.809 | 0.800…0.818 |
| doxazosin | 0.741 | 0.725…0.758 |
| duloxetine | 0.816 | 0.805…0.827 |
| dutasteride | 0.815 | 0.805…0.826 |
| empagliflozin | 0.828 | 0.813…0.843 |
| enalapril | 0.818 | 0.813…0.823 |
| escitalopram | 0.781 | 0.774…0.787 |
| febuxostat | 0.746 | 0.728…0.764 |
| felodipine | 0.845 | 0.833…0.857 |
| fluoxetine | 0.786 | 0.776…0.797 |
| flupenthixol | 0.690 | 0.665…0.714 |
| fluvastatin | 0.759 | 0.731…0.788 |
| formoterol | 0.633 | 0.624…0.642 |
| fosinopril | 0.826 | 0.818…0.833 |
| furosemide | 0.612 | 0.601…0.624 |
| gliclazide | 0.859 | 0.852…0.866 |
| glimepiride | 0.837 | 0.828…0.847 |
| glycopyrronium | 0.755 | 0.734…0.776 |
| haloperidol | 0.633 | 0.614…0.651 |
| hydrochlorothiazide | 0.787 | 0.783…0.791 |
| hydroxychloroquine | 0.767 | 0.752…0.783 |
| indacaterol | 0.754 | 0.731…0.776 |
| indapamide | 0.796 | 0.791…0.801 |
| insulin aspart | 0.727 | 0.716…0.739 |
| insulin detemir | 0.715 | 0.701…0.728 |
| insulin glargine | 0.714 | 0.703…0.726 |
| insulin glulisine, human | 0.712 | 0.681…0.743 |
| insulin lispro | 0.764 | 0.744…0.783 |
| ipratropium | 0.505 | 0.486…0.524 |
| isosorbide | 0.767 | 0.758…0.777 |
| lacidipine | 0.799 | 0.789…0.810 |
| lamotrigine | 0.840 | 0.819…0.861 |
| latanoprost | 0.789 | 0.782…0.796 |
| leflunomide | 0.697 | 0.667…0.728 |
| lercanidipine | 0.836 | 0.828…0.845 |
| letrozole | 0.840 | 0.812…0.868 |
| levetiracetam | 0.899 | 0.875…0.923 |
| levodopa | 0.832 | 0.816…0.849 |
| levothyroxine | 0.883 | 0.879…0.887 |
| linagliptin | 0.844 | 0.832…0.856 |
| lipase | 0.453 | 0.436…0.469 |
| liraglutide | 0.693 | 0.672…0.715 |
| lisinopril | 0.775 | 0.759…0.790 |
| losartan | 0.822 | 0.814…0.830 |
| melperone hydrochloride | 0.724 | 0.708…0.741 |
| mesalamine | 0.699 | 0.672…0.725 |
| metformin | 0.795 | 0.791…0.799 |
| methimazole | 0.772 | 0.755…0.788 |
| methotrexate | 0.882 | 0.871…0.893 |
| methylphenidate | 0.652 | 0.630…0.675 |
| metoprolol | 0.788 | 0.785…0.791 |
| mirtazapine | 0.733 | 0.721…0.745 |
| montelukast | 0.588 | 0.575…0.602 |
| moxonidine | 0.607 | 0.589…0.626 |
| nafronyl | 0.698 | 0.682…0.714 |
| nebivolol | 0.749 | 0.744…0.754 |
| nifedipine | 0.725 | 0.705…0.745 |
| nitrendipine | 0.769 | 0.759…0.779 |
| nortriptyline | 0.696 | 0.670…0.721 |
| olanzapine | 0.803 | 0.786…0.820 |
| olmesartan | 0.786 | 0.778…0.793 |
| oxcarbazepine | 0.770 | 0.743…0.798 |
| paroxetine | 0.751 | 0.738…0.764 |
| pentoxifylline | 0.677 | 0.665…0.690 |
| perindopril | 0.804 | 0.800…0.808 |
| piracetam | 0.593 | 0.569…0.616 |
| pramipexole | 0.802 | 0.773…0.831 |
| pravastatin | 0.770 | 0.741…0.799 |
| promazine | 0.824 | 0.801…0.848 |
| propafenone | 0.723 | 0.711…0.735 |
| propranolol | 0.717 | 0.699…0.735 |
| protease | 0.453 | 0.436…0.469 |
| ramipril | 0.800 | 0.796…0.804 |
| rasagiline | 0.857 | 0.835…0.878 |
| risperidone | 0.780 | 0.763…0.796 |
| rivaroxaban | 0.825 | 0.817…0.833 |
| rosuvastatin | 0.761 | 0.756…0.765 |
| saxagliptin | 0.839 | 0.809…0.868 |
| sertraline | 0.790 | 0.781…0.800 |
| simvastatin | 0.780 | 0.773…0.787 |
| sitagliptin | 0.822 | 0.811…0.833 |
| sotalol | 0.814 | 0.798…0.831 |
| spironolactone | 0.732 | 0.724…0.741 |
| sulfasalazine | 0.712 | 0.692…0.731 |
| tafluprost | 0.819 | 0.804…0.834 |
| tamoxifen | 0.874 | 0.854…0.894 |
| tamsulosin | 0.774 | 0.767…0.780 |
| telmisartan | 0.791 | 0.787…0.795 |
| theophylline | 0.668 | 0.649…0.687 |
| tianeptine | 0.671 | 0.658…0.684 |
| ticagrelor | 0.883 | 0.871…0.894 |
| timolol | 0.803 | 0.797…0.809 |
| tiotropium | 0.797 | 0.782…0.811 |
| torsemide | 0.646 | 0.638…0.653 |
| trandolapril | 0.807 | 0.787…0.827 |
| travoprost | 0.797 | 0.786…0.808 |
| trihexyphenidyl | 0.744 | 0.722…0.766 |
| trimetazidine dihydrochloride | 0.622 | 0.614…0.631 |
| ursodeoxycholate | 0.678 | 0.648…0.707 |
| valproate | 0.810 | 0.794…0.826 |
| valsartan | 0.805 | 0.795…0.815 |
| venlafaxine | 0.846 | 0.835…0.856 |
| verapamil | 0.831 | 0.820…0.841 |
| vildagliptin | 0.826 | 0.806…0.845 |
| vortioxetine | 0.805 | 0.783…0.827 |
| warfarin | 0.922 | 0.917…0.926 |
