# Supplementary material for: Systematic evaluation of medication adherence determinants across 137 active substances on population-level real-world health data
Source: Commun Med (Lond). 2026 Mar 9;6:237. doi: 10.1038/s43856-026-01515-8 (PMC13103320; doi:10.1038/s43856-026-01515-8)
Supplement: Supplementary file 6 — Supplementary Data 3. Average age by ingredient [file 43856_2026_1515_MOESM6_ESM.docx]

**Supplementary Data 3.** Average age by ingredient

| Ingredient | Average age | 95% CI |
| --- | --- | --- |
| agomelatine | 47.1 | 46.0...48.1 |
| albuterol | 30.7 | 29.9...31.5 |
| alendronate | 72.4 | 71.8...72.9 |
| alfuzosin | 55.5 | 54.5...56.5 |
| allopurinol | 67.2 | 66.8...67.6 |
| amiodarone | 71.4 | 70.8...72.1 |
| amlodipine | 66.8 | 66.6...67.0 |
| amylase | 63.8 | 62.8...64.8 |
| anastrozole | 68.5 | 66.9...70.1 |
| apixaban | 75.7 | 75.2...76.2 |
| aripiprazole | 35.9 | 33.9...37.8 |
| atenolol | 68.4 | 67.1...69.7 |
| atorvastatin | 67.7 | 67.4...68.0 |
| benserazide | 76.3 | 75.5...77.1 |
| betaxolol | 72.0 | 70.8...73.2 |
| bicalutamide | 74.5 | 73.8...75.3 |
| bimatoprost | 71.8 | 71.0...72.7 |
| bisoprolol | 67.4 | 66.7...68.2 |
| brinzolamide | 73.2 | 72.7...73.8 |
| bupropion | 40.5 | 39.4...41.6 |
| candesartan | 63.1 | 62.5...63.7 |
| carbamazepine | 57.3 | 56.2...58.4 |
| carvedilol | 68.9 | 67.7...70.0 |
| chlorprothixene | 57.5 | 55.6...59.3 |
| citalopram | 54.9 | 53.8...56.1 |
| clopidogrel | 71.0 | 70.5...71.4 |
| clozapine | 53.5 | 51.4...55.5 |
| cyclosporine | 49.7 | 46.8...52.6 |
| dabigatran | 73.0 | 72.2...73.7 |
| digoxin | 77.1 | 76.7...77.5 |
| donepezil | 77.4 | 76.5...78.3 |
| dorzolamide | 73.7 | 73.1...74.3 |
| doxazosin | 68.8 | 67.8...69.7 |
| duloxetine | 51.0 | 50.1...51.9 |
| dutasteride | 71.7 | 71.2...72.2 |
| empagliflozin | 62.4 | 61.7...63.2 |
| enalapril | 70.1 | 69.8...70.5 |
| escitalopram | 45.7 | 45.2...46.2 |
| febuxostat | 66.7 | 65.7...67.7 |
| felodipine | 72.0 | 71.2...72.8 |
| fluoxetine | 47.1 | 46.2...47.9 |
| flupenthixol | 56.2 | 54.5...58.0 |
| fluvastatin | 65.6 | 64.0...67.2 |
| formoterol | 54.9 | 54.1...55.6 |
| fosinopril | 68.8 | 68.3...69.3 |
| furosemide | 74.0 | 73.4...74.6 |
| gliclazide | 69.3 | 68.7...69.8 |
| glimepiride | 67.5 | 66.9...68.0 |
| glycopyrronium | 68.1 | 67.1...69.1 |
| haloperidol | 69.3 | 68.1...70.4 |
| hydrochlorothiazide | 68.4 | 68.2...68.7 |
| hydroxychloroquine | 50.6 | 49.1...52.1 |
| indacaterol | 68.6 | 67.6...69.7 |
| indapamide | 65.2 | 64.9...65.4 |
| insulin aspart | 58.8 | 57.6...60.0 |
| insulin detemir | 58.5 | 57.3...59.7 |
| insulin glargine | 60.5 | 59.6...61.5 |
| insulin glulisine, human | 48.3 | 45.0...51.7 |
| insulin lispro | 57.5 | 55.4...59.6 |
| ipratropium | 67.3 | 66.3...68.3 |
| isosorbide | 77.3 | 76.9...77.7 |
| lacidipine | 71.6 | 70.9...72.2 |
| lamotrigine | 39.9 | 37.9...41.9 |
| latanoprost | 72.8 | 72.4...73.2 |
| leflunomide | 59.5 | 57.9...61.1 |
| lercanidipine | 69.4 | 68.8...69.9 |
| letrozole | 65.0 | 63.5...66.6 |
| levetiracetam | 31.7 | 28.1...35.2 |
| levodopa | 76.2 | 75.4...77.0 |
| levothyroxine | 59.3 | 58.8...59.8 |
| linagliptin | 69.3 | 68.5...70.0 |
| lipase | 63.8 | 62.8...64.8 |
| liraglutide | 60.7 | 59.6...61.8 |
| lisinopril | 65.1 | 64.2...66.0 |
| losartan | 67.6 | 67.1...68.2 |
| melperone hydrochloride | 73.0 | 71.8...74.2 |
| mesalamine | 46.1 | 43.9...48.2 |
| metformin | 65.6 | 65.3...65.9 |
| methimazole | 58.2 | 56.9...59.6 |
| methotrexate | 54.4 | 53.2...55.6 |
| methylphenidate | 13.6 | 12.9...14.3 |
| metoprolol | 68.7 | 68.5...68.9 |
| mirtazapine | 52.7 | 51.9...53.6 |
| montelukast | 23.4 | 22.2...24.5 |
| moxonidine | 69.0 | 68.2...69.7 |
| nafronyl | 72.2 | 71.4...72.9 |
| nebivolol | 59.5 | 59.1...59.8 |
| nifedipine | 74.8 | 74.0...75.6 |
| nitrendipine | 74.7 | 74.2...75.2 |
| nortriptyline | 60.5 | 58.3...62.7 |
| olanzapine | 48.7 | 47.3...50.1 |
| olmesartan | 64.8 | 64.4...65.2 |
| oxcarbazepine | 42.7 | 40.0...45.5 |
| paroxetine | 49.4 | 48.4...50.4 |
| pentoxifylline | 71.3 | 70.6...72.0 |
| perindopril | 64.3 | 64.0...64.5 |
| piracetam | 64.0 | 62.4...65.6 |
| pramipexole | 64.8 | 63.0...66.7 |
| pravastatin | 73.9 | 72.3...75.5 |
| promazine | 57.5 | 55.7...59.4 |
| propafenone | 69.3 | 68.8...69.9 |
| propranolol | 54.7 | 53.3...56.0 |
| protease | 63.8 | 62.8...64.8 |
| ramipril | 68.4 | 68.2...68.7 |
| rasagiline | 74.0 | 72.8...75.2 |
| risperidone | 55.5 | 53.6...57.5 |
| rivaroxaban | 72.4 | 71.9...72.8 |
| rosuvastatin | 65.8 | 65.5...66.1 |
| saxagliptin | 63.1 | 61.4...64.8 |
| sertraline | 42.9 | 42.1...43.8 |
| simvastatin | 70.4 | 70.0...70.8 |
| sitagliptin | 65.7 | 65.0...66.3 |
| sotalol | 71.1 | 70.2...72.1 |
| spironolactone | 73.5 | 73.1...74.0 |
| sulfasalazine | 54.2 | 52.9...55.5 |
| tafluprost | 69.2 | 68.3...70.2 |
| tamoxifen | 56.1 | 54.3...57.9 |
| tamsulosin | 69.2 | 68.8...69.6 |
| telmisartan | 64.7 | 64.5...65.0 |
| theophylline | 74.0 | 73.2...74.9 |
| tianeptine | 53.6 | 52.8...54.5 |
| ticagrelor | 65.6 | 64.8...66.4 |
| timolol | 72.0 | 71.6...72.4 |
| tiotropium | 65.2 | 64.4...66.0 |
| torsemide | 75.5 | 75.2...75.8 |
| trandolapril | 63.9 | 62.6...65.2 |
| travoprost | 73.1 | 72.5...73.8 |
| trihexyphenidyl | 58.3 | 56.7...59.9 |
| trimetazidine dihydrochloride | 69.0 | 68.6...69.5 |
| ursodeoxycholate | 55.3 | 53.3...57.3 |
| valproate | 40.4 | 38.8...42.0 |
| valsartan | 65.5 | 64.8...66.1 |
| venlafaxine | 45.0 | 44.1...45.9 |
| verapamil | 69.2 | 68.5...69.9 |
| vildagliptin | 62.8 | 61.6...64.0 |
| vortioxetine | 43.0 | 41.6...44.4 |
| warfarin | 71.1 | 70.7...71.5 |
