# Supplementary material for: Systematic evaluation of medication adherence determinants across 137 active substances on population-level real-world health data
Source: Commun Med (Lond). 2026 Mar 9;6:237. doi: 10.1038/s43856-026-01515-8 (PMC13103320; doi:10.1038/s43856-026-01515-8)
Supplement: Supplementary file 7 — Supplementary Data 4. Linear mixed model estimates [file 43856_2026_1515_MOESM7_ESM.docx]

# **Supplementary Data 4.** Estimates of demographic, health- and medication-related variables on CMA in linear mixed model

| **Variable** | **Estimate** | **SE** | **95% CI** | **P-value** | **Sig^1^** |
| --- | --- | --- | --- | --- | --- |
| Intercept | 0.660 | 0.004 | 0.652...0.668 | 0.000 | * |
| Gender (Ref: Female) |  |  |  |  |  |
| Male | -0.001 | 0.001 | -0.004...0.001 | 0.229 |  |
| Age (Ref: 0-19 years) |  |  |  |  |  |
| 20-39 years | 0.081 | 0.003 | 0.075...0.087 | 0.000 | * |
| 40-59 years | 0.079 | 0.003 | 0.073...0.085 | 0.000 | * |
| 60-79 years | 0.098 | 0.003 | 0.091...0.104 | 0.000 | * |
| 80+ years | 0.107 | 0.003 | 0.100...0.113 | 0.000 | * |
| Body mass index (Ref: obese) |  |  |  |  |  |
| BMI: Overweight | -0.010 | 0.002 | -0.014...-0.006 | 0.000 | * |
| BMI: Under or normal weight | -0.009 | 0.002 | -0.014...-0.005 | 0.000 | * |
| BMI: Unknown | -0.011 | 0.002 | -0.014...-0.008 | 0.000 | * |
| Year of administation (Ref: year 1) |  |  |  |  |  |
| Year 2 | 0.043 | 0.001 | 0.041...0.044 | 0.000 | * |
| Year 3 | 0.056 | 0.001 | 0.055...0.058 | 0.000 | * |
| Year 4 | 0.064 | 0.001 | 0.062...0.065 | 0.000 | * |
| Year 5 | 0.067 | 0.001 | 0.065...0.069 | 0.000 | * |
| Year 6 | 0.070 | 0.001 | 0.068...0.072 | 0.000 | * |
| Year 7 | 0.076 | 0.001 | 0.074...0.078 | 0.000 | * |
| Hospitalisation (Ref: No) | 0.009 | 0.001 | 0.007...0.010 | 0.000 | * |
| Depression (Ref: No) | 0.005 | 0.001 | 0003...0.007 | 0.000 | * |
| Dementia or retardation (Ref: No) | 0.004 | 0.002 | 0.000...0.009 | 0.053 |  |
| Comorbidity (Ref: No) | 0.000 | 0.001 | -0.002...0.002 | 0.851 |  |
| Number of chronic ingredients | 0.001 | 0.000 | 0.001...0.002 | 0.000 | * |
| Number of diagnoses | 0.000 | 0.000 | 0.000...0.000 | 0.004 |  |
| Administration route |  |  |  |  |  |
| Inhalation | 0.137 | 0.006 | 0.124...0.149 | 0.000 | * |
| Intramuscular | -0.293 | 0.013 | -0.319...-0.268 | 0.000 | * |
| Intravenous | -0.268 | 0.140 | -0.542...0.006 | 0.055 |  |
| Mixed | 0.060 | 0.007 | 0.047...0.073 | 0.000 | * |
| Ocular | 0.026 | 0.014 | -0.001...0.053 | 0.062 |  |
| Rectal | -0.213 | 0.031 | -0.274...-0.152 | 0.000 | * |
| Subcutaneous | 0.011 | 0.058 | -0.104...0.125 | 0.857 |  |
| Ingredient (Ref: Metoprolol) |  |  |  |  |  |
| Agomelatine | 0.021 | 0.008 | 0.005...0.038 | 0.010 |  |
| Albuterol | -0.393 | 0.006 | -0.405...-0.382 | 0.000 | * |
| Alendronate | -0.048 | 0.019 | -0.085...-0.010 | 0.012 |  |
| Alfuzosin | -0.014 | 0.011 | -0.036...0.008 | 0.210 |  |
| Allopurinol | -0.048 | 0.006 | -0.060...-0.036 | 0.000 | * |
| Amiodarone | -0.024 | 0.008 | -0.040...-0.008 | 0.004 |  |
| Amlodipine | 0.010 | 0.004 | 0.001...0.018 | 0.030 |  |
| Amylase | -0.274 | 0.006 | -0.286...-0.263 | 0.000 | * |
| Anastrozole | -0.183 | 0.021 | -0.224...-0.142 | 0.000 | * |
| Apixaban | 0.027 | 0.011 | 0.005...0.050 | 0.017 |  |
| Aripiprazole | -0.011 | 0.010 | -0.031...0.008 | 0.251 |  |
| Atenolol | -0.026 | 0.017 | -0.060...0.007 | 0.121 |  |
| Atorvastatin | 0.011 | 0.003 | 0.005...0.017 | 0.000 | * |
| Benserazide | -0.048 | 0.016 | -0.079...-0.017 | 0.003 |  |
| Betaxolol | -0.187 | 0.075 | -0.333...-0.041 | 0.012 |  |
| Bicalutamide | 0.001 | 0.117 | -0.228...0.231 | 0.990 |  |
| Bimatoprost | 0.045 | 0.218 | -0.383...0.472 | 0.838 |  |
| Bisoprolol | 0.000 | 0.006 | -0.012...0.012 | 0.982 |  |
| Brinzolamide | -0.356 | 0.089 | -0.531...-0.181 | 0.000 | * |
| Bupropion | 0.032 | 0.009 | 0.014...0.050 | 0.000 | * |
| Candesartan | -0.075 | 0.012 | -0.098...-0.053 | 0.000 | * |
| Carbamazepine | -0.106 | 0.006 | -0.118...-0.094 | 0.000 | * |
| Carvedilol | -0.003 | 0.011 | -0.024...0.019 | 0.817 |  |
| Chlorprothixene | 0.033 | 0.008 | 0.018...0.048 | 0.000 | * |
| Citalopram | -0.021 | 0.006 | -0.033...-0.008 | 0.001 |  |
| Clopidogrel | 0.042 | 0.004 | 0.034...0.050 | 0.000 | * |
| Clozapine | -0.012 | 0.008 | -0.028...0.004 | 0.133 |  |
| Cyclosporine | 0.037 | 0.016 | 0.005...0.069 | 0.023 |  |
| Dabigatran | -0.067 | 0.010 | -0.087...-0.046 | 0.000 | * |
| Digoxin | 0.012 | 0.010 | -0.008...0.031 | 0.233 |  |
| Donepezil | 0.024 | 0.012 | 0.001...0.046 | 0.043 |  |
| Dorzolamide | -0.081 | 0.041 | -0.162...-0.001 | 0.047 |  |
| Doxazosin | -0.041 | 0.009 | -0.059...-0.022 | 0.000 | * |
| Duloxetine | 0.039 | 0.005 | 0.029...0.049 | 0.000 | * |
| Dutasteride | 0.016 | 0.014 | -0.012...0.044 | 0.267 |  |
| Empagliflozin | -0.153 | 0.076 | -0.301...-0.005 | 0.043 |  |
| Enalapril | 0.019 | 0.007 | 0.005...0.032 | 0.007 |  |
| Escitalopram | 0.009 | 0.004 | 0.001...0.017 | 0.027 |  |
| Febuxostat | 0.003 | 0.017 | -0.031...0.036 | 0.878 |  |
| Felodipine | 0.012 | 0.020 | -0.026...0.050 | 0.542 |  |
| Fluoxetine | 0.007 | 0.005 | -0.004...0.017 | 0.208 |  |
| Flupenthixol | -0.073 | 0.008 | -0.090...-0.057 | 0.000 | * |
| Fluvastatin | -0.031 | 0.013 | -0.055...-0.006 | 0.016 |  |
| Formoterol | -0.237 | 0.009 | -0.255...-0.218 | 0.000 | * |
| Fosinopril | 0.010 | 0.009 | -0.008...0.027 | 0.285 |  |
| Furosemide | -0.151 | 0.008 | -0.166...-0.136 | 0.000 | * |
| Gliclazide | 0.046 | 0.024 | -0.001...0.092 | 0.053 |  |
| Glimepiride | 0.032 | 0.037 | -0.042...0.105 | 0.395 |  |
| Glycopyrronium | -0.189 | 0.012 | -0.213...-0.165 | 0.000 | * |
| Haloperidol | -0.131 | 0.005 | -0.142...-0.121 | 0.000 | * |
| Hydrochlorothiazide | -0.023 | 0.005 | -0.032...-0.013 | 0.000 | * |
| Hydroxychloroquine | -0.004 | 0.007 | -0.017...0.010 | 0.595 |  |
| Indacaterol | -0.195 | 0.013 | -0.220...-0.170 | 0.000 | * |
| Indapamide | 0.018 | 0.006 | 0.007...0.029 | 0.002 |  |
| Insulin aspart | -0.060 | 0.059 | -0.176...0.056 | 0.310 |  |
| Insulin detemir | -0.091 | 0.059 | -0.207...0.025 | 0.123 |  |
| Insulin glargine | -0.102 | 0.059 | -0.218...0.014 | 0.084 |  |
| Insulin glulisine, human | -0.060 | 0.060 | -0.177...0.058 | 0.319 |  |
| Insulin lispro | -0.081 | 0.059 | -0.198...0.035 | 0.172 |  |
| Ipratropium | -0.443 | 0.012 | -0.467...-0.419 | 0.000 | * |
| Isosorbide | -0.031 | 0.005 | -0.041...-0.020 | 0.000 | * |
| Lacidipine | 0.013 | 0.017 | -0.021...0.046 | 0.456 |  |
| Lamotrigine | 0.004 | 0.008 | -0.013...0.020 | 0.642 |  |
| Latanoprost | -0.059 | 0.054 | -0.165...0.047 | 0.277 |  |
| Leflunomide | -0.090 | 0.009 | -0.109...-0.072 | 0.000 | * |
| Lercanidipine | 0.049 | 0.011 | 0.026...0.071 | 0.000 | * |
| Letrozole | -0.193 | 0.019 | -0.231...-0.155 | 0.000 | * |
| Levetiracetam | 0.012 | 0.012 | -0.012...0.036 | 0.325 |  |
| Levodopa | -0.046 | 0.016 | -0.077...-0.015 | 0.004 |  |
| Levothyroxine | 0.116 | 0.004 | 0.107...0.124 | 0.000 | * |
| Linagliptin | -0.073 | 0.063 | -0.195...0.050 | 0.246 |  |
| Lipase | -0.274 | 0.006 | -0.286...-0.263 | 0.000 | * |
| Lisinopril | 0.005 | 0.024 | -0.043...0.052 | 0.840 |  |
| Losartan | 0.034 | 0.012 | 0.009...0.058 | 0.007 |  |
| Melperone hydrochloride | -0.055 | 0.006 | -0.067...-0.042 | 0.000 | * |
| Mesalamine | -0.104 | 0.016 | -0.135...-0.073 | 0.000 | * |
| Metformin | -0.035 | 0.007 | -0.048...-0.022 | 0.000 | * |
| Methimazole | -0.026 | 0.027 | -0.078...0.027 | 0.343 |  |
| Methotrexate | 0.098 | 0.007 | 0.085...0.111 | 0.000 | * |
| Methylphenidate | 0.008 | 0.071 | -0.131...0.148 | 0.906 |  |
| Mirtazapine | -0.044 | 0.005 | -0.054...-0.035 | 0.000 | * |
| Montelukast | -0.141 | 0.008 | -0.157...-0.125 | 0.000 | * |
| Moxonidine | -0.079 | 0.010 | -0.098...-0.060 | 0.000 | * |
| Nafronyl | -0.073 | 0.007 | -0.085...-0.060 | 0.000 | * |
| Nebivolol | -0.010 | 0.006 | -0.022...0.001 | 0.070 |  |
| Nifedipine | 0.014 | 0.017 | -0.019...0.048 | 0.403 |  |
| Nitrendipine | -0.021 | 0.011 | -0.042...-0.001 | 0.044 |  |
| Nortriptyline | -0.092 | 0.009 | -0.110...-0.075 | 0.000 | * |
| Olanzapine | 0.008 | 0.006 | -0.004...0.021 | 0.183 |  |
| Olmesartan | 0.017 | 0.010 | -0.002...0.036 | 0.072 |  |
| Oxcarbazepine | -0.069 | 0.009 | -0.087...-0.050 | 0.000 | * |
| Paroxetine | -0.027 | 0.006 | -0.038...-0.016 | 0.000 | * |
| Pentoxifylline | -0.102 | 0.006 | -0.114...-0.091 | 0.000 | * |
| Perindopril | 0.024 | 0.006 | 0.013...0.035 | 0.000 | * |
| Piracetam | -0.122 | 0.008 | -0.137...-0.106 | 0.000 | * |
| Pramipexole | -0.002 | 0.013 | -0.027...0.023 | 0.873 |  |
| Pravastatin | -0.041 | 0.017 | -0.074...-0.008 | 0.016 |  |
| Promazine | 0.022 | 0.008 | 0.006...0.037 | 0.006 |  |
| Propafenone | -0.060 | 0.007 | -0.074...-0.045 | 0.000 | * |
| Propranolol | -0.040 | 0.007 | -0.054...-0.025 | 0.000 | * |
| Protease | -0.274 | 0.006 | -0.286...-0.263 | 0.000 | * |
| Ramipril | 0.013 | 0.004 | 0.005...0.021 | 0.001 |  |
| Rasagiline | -0.022 | 0.017 | -0.055...0.011 | 0.184 |  |
| Risperidone | -0.021 | 0.006 | -0.033...-0.009 | 0.001 |  |
| Rivaroxaban | 0.026 | 0.006 | 0.015...0.037 | 0.000 | * |
| Rosuvastatin | -0.007 | 0.003 | -0.013...0.000 | 0.036 |  |
| Saxagliptin | -0.077 | 0.072 | -0.218...0.065 | 0.289 |  |
| Sertraline | 0.023 | 0.005 | 0.013...0.032 | 0.000 | * |
| Simvastatin | -0.011 | 0.004 | -0.019...-0.004 | 0.004 |  |
| Sitagliptin | -0.083 | 0.042 | -0.166...0.000 | 0.050 |  |
| Sotalol | 0.029 | 0.008 | 0.013...0.045 | 0.000 | * |
| Spironolactone | -0.054 | 0.005 | -0.064...-0.043 | 0.000 | * |
| Sulfasalazine | -0.070 | 0.008 | -0.085...-0.054 | 0.000 | * |
| Tafluprost | -0.338 | 0.072 | -0.480...-0.196 | 0.000 | * |
| Tamoxifen | -0.143 | 0.018 | -0.178...-0.108 | 0.000 | * |
| Tamsulosin | -0.008 | 0.006 | -0.020...0.004 | 0.184 |  |
| Telmisartan | 0.013 | 0.005 | 0.002...0.023 | 0.018 |  |
| Theophylline | -0.152 | 0.009 | -0.170...-0.134 | 0.000 | * |
| Tianeptine | -0.094 | 0.005 | -0.104...-0.083 | 0.000 | * |
| Ticagrelor | 0.028 | 0.008 | 0.011...0.044 | 0.001 |  |
| Timolol | -0.138 | 0.035 | -0.206...-0.069 | 0.000 | * |
| Tiotropium | -0.162 | 0.011 | -0.185...-0.140 | 0.000 | * |
| Torsemide | -0.138 | 0.004 | -0.146...-0.129 | 0.000 | * |
| Trandolapril | 0.034 | 0.025 | -0.014...0.083 | 0.167 |  |
| Travoprost | -0.371 | 0.072 | -0.512...-0.229 | 0.000 | * |
| Trihexyphenidyl | -0.056 | 0.007 | -0.069...-0.043 | 0.000 | * |
| Trimetazidine dihydrochloride | -0.127 | 0.004 | -0.135...-0.118 | 0.000 | * |
| Ursodeoxycholate | -0.138 | 0.014 | -0.165...-0.111 | 0.000 | * |
| Valproate | -0.026 | 0.006 | -0.038...-0.013 | 0.000 | * |
| Valsartan | 0.021 | 0.013 | -0.003...0.046 | 0.089 |  |
| Venlafaxine | 0.064 | 0.006 | 0.053...0.075 | 0.000 | * |
| Verapamil | 0.037 | 0.008 | 0.021...0.053 | 0.000 | * |
| Vildagliptin | -0.161 | 0.101 | -0.359...0.036 | 0.110 |  |
| Vortioxetine | 0.038 | 0.011 | 0.016...0.060 | 0.001 |  |
| Warfarin | 0.120 | 0.004 | 0.112...0.129 | 0.000 | * |
| Disease (Ref: No) |  |  |  |  |  |
| Malignant neoplasm of breast (C50) | 0.300 | 0.019 | 0.263...0.337 | 0.000 | * |
| Malignant neoplasm of prostate  (C61) | 0.022 | 0.117 | -0.208...0.252 | 0.853 |  |
| Other hypothyroidism (E03) | -0.016 | 0.004 | -0.024...-0.008 | 0.000 | * |
| Hyperthyroidism (E05) | 0.021 | 0.028 | -0.033...0.075 | 0.445 |  |
| Type 1 diabetes mellitus (E10) | 0.005 | 0.009 | -0.013...0.022 | 0.596 |  |
| Type 2 diabetes mellitus (E11) | 0v203 | 0.101 | 0.005...0.401 | 0.044 |  |
| Disorders of lipoprotein metabolism and other lipidaemias (E78) | -0.004 | 0.004 | -0.012...0.004 | 0.347 |  |
| Dementia in Alzheimer disease (F00) | -0.004 | 0.017 | -0.037...0.030 | 0.837 |  |
| Vascular dementia (F01) | 0.001 | 0.010 | -0.020...0.021 | 0.958 |  |
| Other mental disorders due to brain damage and dysfunction and to physical disease (F06) | 0.009 | 0.011 | -0.013...0.031 | 0.435 |  |
| Schizophrenia (F20) | 0.029 | 0.005 | 0.019...0.040 | 0.000 | * |
| Persistent delusional disorders (F22) | 0.031 | 0.019 | -0.007...0.069 | 0.109 |  |
| Depressive episode (F32) | -0.003 | 0.020 | -0.041...0.035 | 0.881 |  |
| Recurrent depressive disorder (F33) | 0.002 | 0.003 | -0.005...0.008 | 0.641 |  |
| Other anxiety disorders (F41) | 0.015 | 0.003 | 0.009...0.021 | 0.000 | * |
| Hyperkinetic disorders (F90) | -0.048 | 0.072 | -0.189...0.093 | 0.503 |  |
| Parkinson disease (G20) | 0.087 | 0.015 | 0.057...0.117 | 0.000 | * |
| Other extrapyramidal and movement disorders (G25) | -0.003 | 0.012 | -0.026...0.021 | 0.830 |  |
| Alzheimer disease (G30) | 0.004 | 0.021 | -0.037...0.044 | 0.853 |  |
| Epilepsy (G40) | 0.095 | 0.006 | 0.084...0.107 | 0.000 | * |
| Glaucoma (H40) | 0.349 | 0.071 | 0.210...0.488 | 0.000 | * |
| Essential (primary) hypertension (I10) | 0.006 | 0.002 | 0.001...0.010 | 0.022 |  |
| Hypertensive heart disease (I11) | 0.017 | 0.002 | 0.013...0.021 | 0.000 | * |
| Angina pectoris (I20) | 0.015 | 0.005 | 0.005...0.024 | 0.002 |  |
| Chronic ischaemic heart disease (I25) | 0.035 | 0.007 | 0.022...0.049 | 0.000 | * |
| Atrial fibrillation and flutter (I48) | -0.011 | 0.005 | -0.02...-0.002 | 0.020 |  |
| Other cardiac arrhythmias (I49) | -0.012 | 0.007 | -0.025...0.001 | 0.075 |  |
| Heart failure (I50) | 0.014 | 0.004 | 0.006...0.023 | 0.001 |  |
| Other cerebrovascular diseases (I67) | -0.049 | 0.016 | -0.081...-0.018 | 0.002 |  |
| Atherosclerosis (I70) | 0.005 | 0.006 | -0.007...0.017 | 0.414 |  |
| Other chronic obstructive pulmonary disease (J44) | 0.037 | 0.008 | 0.021...0.054 | 0.000 | * |
| Asthma (J45) | 0.061 | 0.011 | 0.039...0.082 | 0.000 | * |
| Gastritis and duodenitis (K29) | -0.008 | 0.007 | -0.022...0.006 | 0.270 |  |
| Functional dyspepsia (K30) | -0.050 | 0.008 | -0.065...-0.036 | 0.000 | * |
| Ulcerative colitis (K51) | 0.050 | 0.018 | 0.014...0.086 | 0.006 |  |
| Chronic hepatitis, not elsewhere classified (K73) | 0.059 | 0.023 | 0.014...0.104 | 0.011 |  |
| Fibrosis and cirrhosis of liver (K74) | 0.113 | 0.019 | 0.075...0.151 | 0.000 | * |
| Cholelithiasis (K80) | 0.053 | 0.027 | 0.000...0.105 | 0.048 |  |
| Other diseases of pancreas (K86) | -0.002 | 0.007 | -0.015...0.011 | 0.748 |  |
| Seropositive rheumatoid arthritis (M05) | 0.025 | 0.007 | 0.011...0.039 | 0.000 | * |
| Other rheumatoid arthritis (M06) | 0.024 | 0.007 | 0.010...0.038 | 0.001 | * |
| Psoriatic and enteropathic arthropathies (M07) | 0.016 | 0.012 | -0.009...0.040 | 0.206 |  |
| Gout (M10) | -0.056 | 0.018 | -0.091...-0.021 | 0.002 |  |
| Other inflammatory spondylopathies (M46) | -0.033 | 0.016 | -0.066...-0.001 | 0.043 |  |
| Osteoporosis with pathological fracture (M80) | 0.086 | 0.019 | 0.049...0.123 | 0.000 | * |
| Osteoporosis without pathological fracture (M81) | 0.060 | 0.019 | 0.023...0.097 | 0.001 |  |
| Hyperplasia of prostate (N40) | 0.018 | 0.006 | 0.006...0.030 | 0.004 |  |
| Inflammatory diseases of prostate (N41) | -0.027 | 0.013 | -0.052...-0.002 | 0.037 |  |
| Failure and rejection of transplanted organs and tissues (T86) | 0.099 | 0.022 | 0.057...0.142 | 0.000 | * |
| Transplanted organ and tissue status (Z94) | 0.077 | 0.024 | 0.030...0.123 | 0.001 |  |
| Presence of cardiac and vascular implants and grafts (Z95) | 0.061 | 0.006 | 0.050...0.072 | 0.000 | * |
| Ingredient x Disease |  |  |  |  |  |
| Amlodipine:Hypertensive heart disease (I11) | -0.006 | 0.004 | -0.015...0.003 | 0.186 |  |
| Atenolol:Hypertensive heart disease (I11) | 0.026 | 0.018 | -0.010...0.062 | 0.153 |  |
| Bisoprolol:Hypertensive heart disease (I11) | -0.020 | 0.008 | -0.035...-0.005 | 0.009 |  |
| Candesartan:Hypertensive heart disease (I11) | 0.013 | 0.012 | -0.010...0.035 | 0.274 |  |
| Carvedilol:Hypertensive heart disease (I11) | -0.042 | 0.014 | -0.069...-0.014 | 0.003 |  |
| Doxazosin:Hypertensive heart disease (I11) | -0.026 | 0.012 | -0.050...-0.003 | 0.026 |  |
| Enalapril:Hypertensive heart disease (I11) | 0.002 | 0.007 | -0.012...0.015 | 0.786 |  |
| Felodipine:Hypertensive heart disease (I11) | 0.022 | 0.020 | -0.016...0.061 | 0.260 |  |
| Fosinopril:Hypertensive heart disease (I11) | 0.016 | 0.009 | -0.002...0.034 | 0.073 |  |
| Hydrochlorothiazide:Hypertensive heart disease (I11) | 0.020 | 0.005 | 0.011...0.030 | 0.000 | * |
| Indapamide:Hypertensive heart disease (I11) | -0.001 | 0.006 | -0.013...0.010 | 0.794 |  |
| Lacidipine:Hypertensive heart disease (I11) | -0.017 | 0.017 | -0.050...0.016 | 0.314 |  |
| Lercanidipine:Hypertensive heart disease (I11) | -0.010 | 0.012 | -0.032...0.013 | 0.396 |  |
| Lisinopril:Hypertensive heart disease (I11) | -0.002 | 0.024 | -0.050...0.045 | 0.923 |  |
| Losartan:Hypertensive heart disease (I11) | -0.013 | 0.012 | -0.037...0.012 | 0.311 |  |
| Moxonidine:Hypertensive heart disease (I11) | -0.039 | 0.011 | -0.060...-0.018 | 0.000 | * |
| Nebivolol:Hypertensive heart disease (I11) | -0.003 | 0.006 | -0.015...0.008 | 0.597 |  |
| Nifedipine:Hypertensive heart disease (I11) | -0.030 | 0.017 | -0.064...0.004 | 0.083 |  |
| Nitrendipine:Hypertensive heart disease (I11) | 0.007 | 0.011 | -0.014...0.028 | 0.541 |  |
| Olmesartan:Hypertensive heart disease (I11) | -0.006 | 0.009 | -0.025...0.012 | 0.517 |  |
| Perindopril:Hypertensive heart disease (I11) | -0.002 | 0.006 | -0.013...0.009 | 0.765 |  |
| Propranolol:Hypertensive heart disease (I11) | 0.003 | 0.015 | -0.027...0.033 | 0.856 |  |
| Ramipril:Hypertensive heart disease (I11) | -0.003 | 0.004 | -0.011...0.006 | 0.513 |  |
| Telmisartan:Hypertensive heart disease (I11) | -0.010 | 0.005 | -0.021...0.000 | 0.057 |  |
| Trandolapril:Hypertensive heart disease (I11) | -0.014 | 0.025 | -0.062...0.035 | 0.584 |  |
| Trimetazidine dihydrochloride:Hypertensive heart disease (I11) | -0.028 | 0.006 | -0.040...-0.017 | 0.000 | * |
| Valsartan:Hypertensive heart disease (I11) | -0.012 | 0.013 | -0.037...0.012 | 0.324 |  |
| Verapamil:Hypertensive heart disease (I11) | -0.006 | 0.009 | -0.024...0.011 | 0.475 |  |
| Amlodipine:Essential (primary) hypertension (I10) | 0.008 | 0.004 | -0.001...0.017 | 0.073 |  |
| Atenolol:Essential (primary) hypertension (I10) | 0.049 | 0.019 | 0.013...0.086 | 0.008 |  |
| Candesartan:Essential (primary) hypertension (I10) | 0.017 | 0.012 | -0.006...0.040 | 0.147 |  |
| Enalapril:Essential (primary) hypertension (I10) | 0.006 | 0.007 | -0.008...0.020 | 0.380 |  |
| Felodipine:Essential (primary) hypertension (I10) | 0.028 | 0.020 | -0.010...0.067 | 0.152 |  |
| Fosinopril:Essential (primary) hypertension (I10) | 0.013 | 0.009 | -0.005...0.031 | 0.171 |  |
| Hydrochlorothiazide:Essential (primary) hypertension (I10) | 0.025 | 0.005 | 0.015...0.035 | 0.000 | * |
| Indapamide:Essential (primary) hypertension (I10) | 0.005 | 0.006 | -0.007...0.016 | 0.435 |  |
| Lacidipine:Essential (primary) hypertension (I10) | -0.001 | 0.017 | -0.034...0.032 | 0.949 |  |
| Lercanidipine:Essential (primary) hypertension (I10) | -0.002 | 0.012 | -0.025...0.021 | 0.843 |  |
| Lisinopril:Essential (primary) hypertension (I10) | 0.021 | 0.024 | -0.026...0.068 | 0.381 |  |
| Losartan:Essential (primary) hypertension (I10) | -0.009 | 0.012 | -0.033...0.015 | 0.462 |  |
| Nebivolol:Essential (primary) hypertension (I10) | -0.001 | 0.006 | -0.013...0.011 | 0.882 |  |
| Nifedipine:Essential (primary) hypertension (I10) | -0.042 | 0.018 | -0.076...-0.007 | 0.017 |  |
| Nitrendipine:Essential (primary) hypertension (I10) | 0.007 | 0.011 | -0.014...0.029 | 0.501 |  |
| Olmesartan:Essential (primary) hypertension (I10) | -0.005 | 0.009 | -0.023...0.014 | 0.618 |  |
| Perindopril:Essential (primary) hypertension (I10) | 0.004 | 0.006 | -0.007...0.015 | 0.458 |  |
| Propranolol:Essential (primary) hypertension (I10) | -0.020 | 0.015 | -0.049...0.008 | 0.162 |  |
| Ramipril:Essential (primary) hypertension (I10) | 0.001 | 0.005 | -0.007...0.010 | 0.743 |  |
| Telmisartan:Essential (primary) hypertension (I10) | -0.001 | 0.005 | -0.011...0.010 | 0.926 |  |
| Trandolapril:Essential (primary) hypertension (I10) | -0.003 | 0.025 | -0.051...0.046 | 0.918 |  |
| Valsartan:Essential (primary) hypertension (I10) | -0.014 | 0.013 | -0.039...0.011 | 0.267 |  |
| Verapamil:Essential (primary) hypertension (I10) | 0.007 | 0.010 | -0.013...0.027 | 0.475 |  |
| Empagliflozin:Type 2 diabetes mellitus (E11) | -0.007 | 0.126 | -0.254...0.241 | 0.957 |  |
| Gliclazide:Type 2 diabetes mellitus (E11) | -0.193 | 0.104 | -0.396...0.011 | 0.063 |  |
| Glimepiride:Type 2 diabetes mellitus (E11) | -0.190 | 0.108 | -0.401...0.021 | 0.078 |  |
| Insulin aspart:Type 2 diabetes mellitus (E11) | -0.230 | 0.102 | -0.429...-0.031 | 0.023 |  |
| Insulin detemir:Type 2 diabetes mellitus (E11) | -0.204 | 0.102 | -0.403...-0.005 | 0.045 |  |
| Insulin glargine:Type 2 diabetes mellitus (E11) | -0.185 | 0.102 | -0.384...0.015 | 0.069 |  |
| Insulin glulisine, human:Type 2 diabetes mellitus (E11) | -0.251 | 0.103 | -0.452...-0.050 | 0.014 |  |
| Insulin lispro:Type 2 diabetes mellitus (E11) | -0.155 | 0.102 | -0.355...0.045 | 0.130 |  |
| Linagliptin:Type 2 diabetes mellitus (E11) | -0.081 | 0.119 | -0.314...0.152 | 0.497 |  |
| Liraglutide:Type 2 diabetes mellitus (E11) | -0.341 | 0.117 | -0.570...-0.111 | 0.004 |  |
| Metformin:Type 2 diabetes mellitus (E11) | -0.168 | 0.101 | -0.367...0.030 | 0.097 |  |
| Saxagliptin:Type 2 diabetes mellitus (E11) | -0.077 | 0.125 | -0.322...0.167 | 0.535 |  |
| Sitagliptin:Type 2 diabetes mellitus (E11) | -0.099 | 0.110 | -0.314...0.116 | 0.367 |  |
| Betaxolol:Glaucoma (H40) | -0.171 | 0.102 | -0.371...0.029 | 0.093 |  |
| Bimatoprost:Glaucoma (H40) | -0.421 | 0.229 | -0.869...0.028 | 0.066 |  |
| Brinzolamide:Glaucoma (H40) | 0.012 | 0.113 | -0.210...0.234 | 0.915 |  |
| Dorzolamide:Glaucoma (H40) | -0.272 | 0.080 | -0.429...-0.115 | 0.001 | * |
| Latanoprost:Glaucoma (H40) | -0.319 | 0.088 | -0.492...-0.146 | 0.000 | * |
| Timolol:Glaucoma (H40) | -0.219 | 0.077 | -0.370...-0.068 | 0.005 |  |
| Atorvastatin:Disorders of lipoprotein metabolism and other lipidaemias (E78) | -0.011 | 0.005 | -0.021...-0.001 | 0.029 |  |
| Fluvastatin:Disorders of lipoprotein metabolism and other lipidaemias (E78) | 0.034 | 0.017 | 0.002...0.067 | 0.038 |  |
| Pravastatin:Disorders of lipoprotein metabolism and other lipidaemias (E78) | 0.017 | 0.020 | -0.021...0.056 | 0.377 |  |
| Rosuvastatin:Disorders of lipoprotein metabolism and other lipidaemias (E78) | -0.012 | 0.005 | -0.022...-0.002 | 0.019 |  |
| Carvedilol:Heart failure (I50) | 0.012 | 0.013 | -0.015...0.038 | 0.384 |  |
| Digoxin:Heart failure (I50) | -0.009 | 0.011 | -0.030...0.013 | 0.432 |  |
| Furosemide:Heart failure (I50) | -0.018 | 0.009 | -0.036...0.001 | 0.058 |  |
| Spironolactone:Heart failure (I50) | -0.002 | 0.007 | -0.016...0.012 | 0.769 |  |
| Amiodarone:Atrial fibrillation and flutter (I48) | 0.025 | 0.010 | 0.005...0.044 | 0.015 |  |
| Apixaban:Atrial fibrillation and flutter (I48) | 0.017 | 0.013 | -0.007...0.042 | 0.167 |  |
| Dabigatran:Atrial fibrillation and flutter (I48) | 0.085 | 0.012 | 0.061...0.109 | 0.000 | * |
| Propafenone:Atrial fibrillation and flutter (I48) | 0.047 | 0.009 | 0.029...0.065 | 0.000 | * |
| Rivaroxaban:Atrial fibrillation and flutter (I48) | 0.037 | 0.008 | 0.022...0.052 | 0.000 | * |
| Sotalol:Atrial fibrillation and flutter (I48) | 0.026 | 0.011 | 0.003...0.048 | 0.024 |  |
| Verapamil:Atrial fibrillation and flutter (I48) | 0.029 | 0.012 | 0.005...0.053 | 0.016 |  |
| Albuterol:Asthma (J45) | 0.045 | 0.012 | 0.021...0.069 | 0.000 | * |
| Formoterol:Asthma (J45) | -0.079 | 0.013 | -0.105...-0.053 | 0.000 | * |
| Ipratropium:Asthma (J45) | -0.017 | 0.014 | -0.045...0.010 | 0.212 |  |
| Montelukast:Asthma (J45) | 0.027 | 0.014 | -0.001...0.056 | 0.057 |  |
| Alfuzosin:Hyperplasia of prostate (N40) | -0.004 | 0.013 | -0.030...0.023 | 0.786 |  |
| Doxazosin:Hyperplasia of prostate (N40) | -0.007 | 0.012 | -0.032...0.017 | 0.571 |  |
| Dutasteride:Hyperplasia of prostate (N40) | -0.009 | 0.015 | -0.040...0.021 | 0.542 |  |
| Agomelatine:F32 | -0.017 | 0.023 | -0.062...0.028 | 0.457 |  |
| Bupropion:Depressive episode (F32) | 0.001 | 0.023 | -0.044...0.046 | 0.980 |  |
| Citalopram:Depressive episode (F32) | 0.017 | 0.022 | -0.026...0.060 | 0.434 |  |
| Duloxetine:Depressive episode (F32) | 0.018 | 0.022 | -0.024...0.061 | 0.400 |  |
| Escitalopram:Depressive episode (F32) | -0.004 | 0.020 | -0.044...0.035 | 0.833 |  |
| Fluoxetine:Depressive episode (F32) | 0.022 | 0.021 | -0.019...0.063 | 0.297 |  |
| Flupenthixol:Depressive episode (F32) | 0.002 | 0.029 | -0.054...0.058 | 0.951 |  |
| Mirtazapine:Depressive episode (F32) | 0.024 | 0.021 | -0.017...0.065 | 0.254 |  |
| Nortriptyline:Depressive episode (F32) | -0.001 | 0.027 | -0.055...0.053 | 0.965 |  |
| Paroxetine:Depressive episode (F32) | 0.008 | 0.022 | -0.034...0.051 | 0.709 |  |
| Sertraline:Depressive episode (F32) | 0.014 | 0.021 | -0.027...0.055 | 0.497 |  |
| Tianeptine:Depressive episode (F32) | -0.010 | 0.021 | -0.051...0.032 | 0.653 |  |
| Venlafaxine:Depressive episode (F32) | 0.003 | 0.021 | -0.039...0.045 | 0.889 |  |
| Allopurinol:Gout (M10) | 0.052 | 0.019 | 0.014...0.089 | 0.007 |  |

**^1^**Statistically significant after Bonferroni correction
